# Supplementary material for: Development and deployment of a histopathology-based deep learning algorithm for patient prescreening in a clinical trial
Source: Nat Commun. 2024 Jun 1;15:4690. doi: 10.1038/s41467-024-49153-9 (PMC11144215; doi:10.1038/s41467-024-49153-9)
Supplement: Supplementary file 1 — Supplementary Information [file 41467_2024_49153_MOESM1_ESM.pdf]

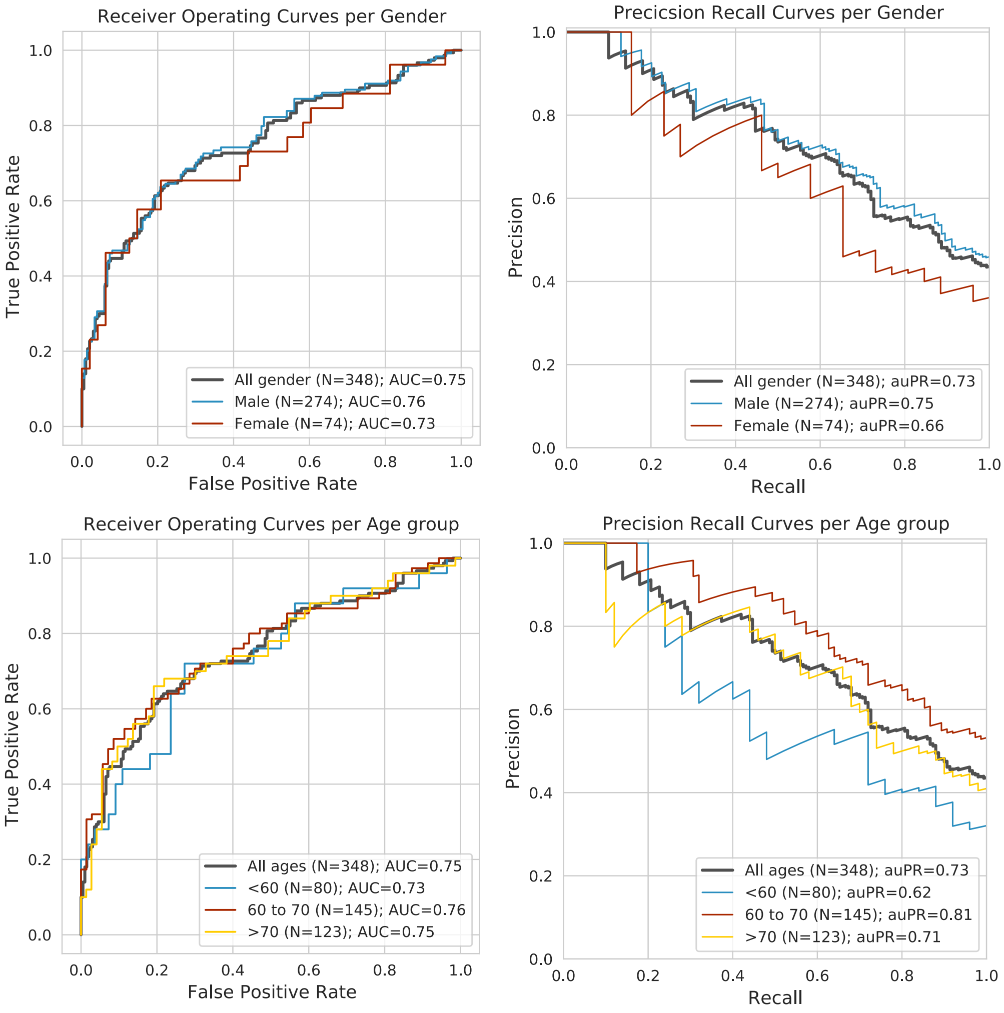

Supplemental Figure 1. Retrospective Validation Performance Stratified by Gender and Age. First row shows Receiver Operating Curves (ROC) and Precision-Recall (PR) curves stratified by gender. Second row shows same curves when stratifying by age. Ages were grouped into 3 groups: <60, 60-70 and >70 years old. Performances are summarized in the legend by area under the ROC curve (AUC) and PR curve (auPR) respectively. Note that the prevalence of *FGFR* in the Retrospective Validation dataset was ~42% (150 *FGFR*+ out of 350 samples).

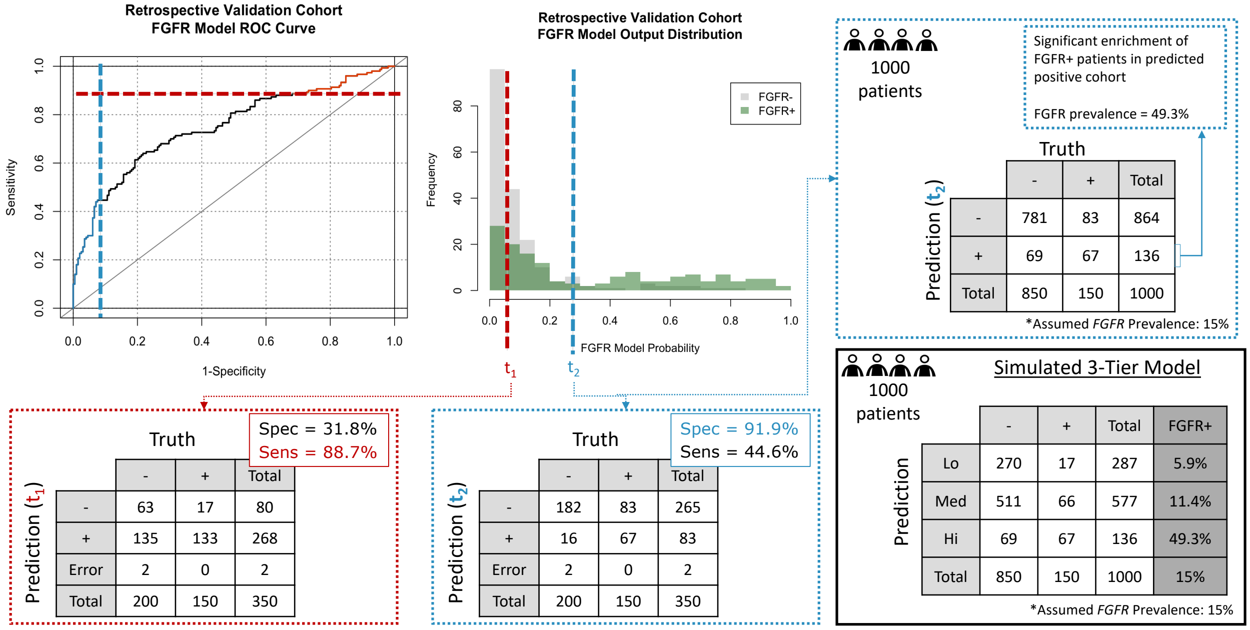

Supplemental Figure 2. Calculation of 3-tier model thresholds. Given the 350 sample Retrospective Validation outputs, we evaluated two thresholds: t1 (red) corresponding to the high sensitivity threshold chosen during model development (i.e., to meet target sensitivity of 0.9 in development data), and t2 (blue) corresponding to the high specificity threshold (to meet target specificity of 0.9 in development data). Shown in the top-left side are the Receiver Operating Curve (ROC) for the Retrospective Validation, along with the model output distribution with the two thresholds for reference. Bottom-left tables show the confusion matrices on the 350 samples for each threshold: t1 (red) and t2 (blue) respectively. Shown in the right side are the simulated confusion matrices given 1,000 patients assuming typical *FGFR*+ prevalence in trial of ~15% [27] for the three tiers given thresholds t1 and t2.
