# Supplementary material for: Development and deployment of a histopathology-based deep learning algorithm for patient prescreening in a clinical trial
Source: Nat Commun. 2024 Jun 1;15:4690. doi: 10.1038/s41467-024-49153-9 (PMC11144215; doi:10.1038/s41467-024-49153-9)
Supplement: Supplementary file 3 — Reporting Summary [file 41467_2024_49153_MOESM3_ESM.pdf]

nature portfolio

Corresponding author(s): Albert Juan Ramon

Last updated by author(s): 4/2/2024

Reporting Summary

Nature Portfolio wishes to improve the reproducibility of the work that we publish. This form provides structure for consistency and transparency in reporting. For further information on Nature Portfolio policies, see our Editorial Policies and the Editorial Policy Checklist.

Statistics

For all statistical analyses, confirm that the following items are present in the figure legend, table legend, main text, or Methods section.

n/a	Confirmed	
☐	☒	The exact sample size (*n*) for each experimental group/condition, given as a discrete number and unit of measurement
☐	☒	A statement on whether measurements were taken from distinct samples or whether the same sample was measured repeatedly
☐	☒	The statistical test(s) used AND whether they are one- or two-sided<br>*Only common tests should be described solely by name; describe more complex techniques in the Methods section.*
☒	☐	A description of all covariates tested
☒	☐	A description of any assumptions or corrections, such as tests of normality and adjustment for multiple comparisons
☐	☒	A full description of the statistical parameters including central tendency (e.g. means) or other basic estimates (e.g. regression coefficient) AND variation (e.g. standard deviation) or associated estimates of uncertainty (e.g. confidence intervals)
☐	☒	For null hypothesis testing, the test statistic (e.g. *F*, *t*, *r*) with confidence intervals, effect sizes, degrees of freedom and *P* value noted<br>*Give P values as exact values whenever suitable.*
☒	☐	For Bayesian analysis, information on the choice of priors and Markov chain Monte Carlo settings
☒	☐	For hierarchical and complex designs, identification of the appropriate level for tests and full reporting of outcomes
☒	☐	Estimates of effect sizes (e.g. Cohen's *d*, Pearson's *r*), indicating how they were calculated

Our web collection on statistics for biologists contains articles on many of the points above.

Software and code

Policy information about availability of computer code

Data collection	We did not use any data collection software
Data analysis	We provide a technical description of the FGFR device in the online Methods, together with supplemental Figure 1 depicting the device structure and pseudocode to facilitate the understanding of the deep learning algorithm for biomarker prediction from H&E WSIs. This includes a description of what sections of the pipeline were based on open-source code, available at https://github.com/CODAIT/deep-histopath/tree/master/deephistopath/wsi. The full code base from the FGFR device is not publicly disclosed to safeguard Janssen R&D intellectual property. Access requests for such code will not be considered to safeguard Johnson & Johnson Innovative Medicine’s intellectual property. However, access to predictions and source code for data analyses and figure generation in this work are publicly available and can be downloaded from https://github.com/johnsonandjohnson/FGFR_Device_Review.

For manuscripts utilizing custom algorithms or software that are central to the research but not yet described in published literature, software must be made available to editors and reviewers. We strongly encourage code deposition in a community repository (e.g. GitHub). See the Nature Portfolio guidelines for submitting code & software for further information.

Data

Policy information about availability of data

All manuscripts must include a data availability statement. This statement should provide the following information, where applicable:

- Accession codes, unique identifiers, or web links for publicly available datasets
- A description of any restrictions on data availability
- For clinical datasets or third party data, please ensure that the statement adheres to our policy

The raw data used in this article was collected from multiple Janssen R&D (Johnson & Johnson) clinical studies (NCT03955913, NCT03390504, NCT03473743) where data was approved for research use. This raw data is not publicly available due to reasons of data sensitivity, including research participant's privacy/consent. Inquiries about clinical study raw data may be made to the authors (ajuanram@its.jnj.com), although access is subject to permission of the corresponding data owners for each of the Janssen R&D (Johnson & Johnson) clinical studies listed above. We also used public raw data from The Cancer Genome Atlas (TCGA) consortium for development. This data is publicly available at https://portal.gdc.cancer.gov/projects/TCGA-BLCA.
The processed source data and code required to reproduce the results presented in this manuscript are publicly available at https://github.com/johnsonandjohnson/FGFR_Device_Review.

Research involving human participants, their data, or biological material

Policy information about studies with human participants or human data. See also policy information about sex, gender (identity/presentation), and sexual orientation and race, ethnicity and racism.

Reporting on sex and gender	Supplemental Figure 1 reports performance stratified by gender and age (grouped)
Reporting on race, ethnicity, or other socially relevant groupings	We stratified analyses by gender and age where these variables were available (i.e., Retrospective Validation data). We did not stratify analyses by race, ethnicity or other groupings a because these variables were not readily available in several datasets.
Population characteristics	Here are the gender distributions considering the samples for which it was available:<br>Development datasets:<br>- TCGA: 295 male, 112 female<br>- BLC3001: 2139 male, 613 female<br>- BLC2002: 147 male, 34 female<br>Retrospective Validation: 275 male, 75 female<br>Full Deployment Data:
Recruitment	Information about clinical study recruitment can be found in https://classic.clinicaltrials.gov/ct2/show/NCT03955913
Ethics oversight	The study was approved by an ethics review boards from sites participating in the ANNAR study (NCT03955913) sponsored by Janssen. The study was carried out in accordance with relevant legislation and ethics guidelines. Enrolled patients provided informed consent prior to participating in the study.

Note that full information on the approval of the study protocol must also be provided in the manuscript.

Field-specific reporting

Please select the one below that is the best fit for your research. If you are not sure, read the appropriate sections before making your selection.

☒ Life sciences ☐ Behavioural & social sciences ☐ Ecological, evolutionary & environmental sciences

For a reference copy of the document with all sections, see nature.com/documents/nr-reporting-summary-flat.pdf

Life sciences study design

All studies must disclose on these points even when the disclosure is negative.

Sample size	The Retrospective Validation dataset was comprised of 350 (150 FGFR+, 200 FGFR-) samples; to achieve a 93% power at detecting a 10% difference in sensitivity using a two-sided exact test with 5% type I error.
Data exclusions	No data was excluded from the analyses.
Replication	The 350 samples used for Retrospective Validation were randomly selected from the entire pool of 3161 samples from BLC3001 (NCT03390504). This data split was performed by an independent software tester, not the developer of the tool, following international standards for device quality management systems (ISO 13485). To ensure the integrity of these samples, the Retrospective Validation was performed after the model had been locked, packaged for deployment and software verification and testing had been performed (under device quality management systems (ISO 13485)). The packaged and verified/tested algorithm was run by an independent software tester on two different environments (QA and Production) to verify that the outputs from the packaged algorithm were the same on both

environments, replicating performance metrics on both. This step was performed a second time after the packaged algorithm was on boarded on the deployment platform as well. All attempts at replication were successful and are recorded in the design control documentation required to label the packaged algorithm as a software as medical device (SaMD)

Randomization	The Development Data was split into Training Data (85%, or 2820 slides) and Hold-out Data (15%, or 582 slides), preserving the same ratio of FGFR+ vs. FGFR- patients, as well as the proportion of samples from each cohort (TCGA, BLC3001 and BLC2002).<br><br>The Retrospective Validation dataset was randomly selected from the entire pool of 3161 samples from BLC3001 (NCT03390504), ensuring a total of 150 FGFR+ and 200 FGFR- were selected; to achieve a 93% power at detecting a 10% difference in sensitivity using a two-sided exact test with 5% type I error.<br><br>There were no patients used in both Development and Retrospective Validation.
Blinding	Investigators were blinded to group allocation and data split during collection and analysis.

Reporting for specific materials, systems and methods

We require information from authors about some types of materials, experimental systems and methods used in many studies. Here, indicate whether each material, system or method listed is relevant to your study. If you are not sure if a list item applies to your research, read the appropriate section before selecting a response.

Materials & experimental systems

n/a	Involved in the study
☒	☐ Antibodies
☒	☐ Eukaryotic cell lines
☒	☐ Palaeontology and archaeology
☒	☐ Animals and other organisms
☐	☒ Clinical data
☒	☐ Dual use research of concern
☒	☐ Plants

Methods

n/a	Involved in the study
☒	☐ ChIP-seq
☒	☐ Flow cytometry
☒	☐ MRI-based neuroimaging

Clinical data

Policy information about clinical studies

All manuscripts should comply with the ICMJE guidelines for publication of clinical research and a completed CONSORT checklist must be included with all submissions.

Clinical trial registration	NCT03955913
Study protocol	https://clinicaltrials.gov/study/NCT03955913?a=23
Data collection	From 2019-08-01 to 2022-09-19
Outcomes	Not applicable, NCT03955913 was an observational non-interventional study. No clinical data was used from other clinical studies.
